# Tumour growth and resistance to gemcitabine of pancreatic cancer cells are decreased by AP-2*α* overexpression

**DOI:** 10.1038/sj.bjc.6605190

**Published:** 2009-08-11

**Authors:** N Jonckheere, V Fauquette, L Stechly, N Saint-Laurent, S Aubert, C Susini, G Huet, N Porchet, I Van Seuningen, P Pigny

**Affiliations:** 1 INSERM, U837, Place de Verdun, 59045 Lille cedex, France; 2 Université de Lille 2, Centre de Recherche Jean-Pierre Aubert, Place de Verdun, 59045 Lille cedex, France; 3 Centre de Biologie et Pathologie, CHRU, 59037 Lille cedex, France; 4 INSERM U858, Institut de Médecine Moléculaire de Rangueil, BP 84225, 31432 Toulouse Cedex 4, France

**Keywords:** mucin, AP-2α, gemcitabine, pancreatic cancer, cell cycle

## Abstract

**Background:**

Activator protein-2*α* (AP-2*α*) is a transcription factor that belongs to the family of AP-2 proteins that have essential roles in tumorigenesis. Indeed, AP-2*α* is considered as a tumour-suppressor gene in different tissues such as colonic, prostatic or breast epithelial cells. Moreover, AP-2*α* also participates in the control of colon and breast cancer cells sensitivity towards chemotherapeutic drugs. Despite its potential interest, very few data are available regarding the roles of AP-2*α* in pancreatic cancer.

**Methods:**

We have developed a stable pancreatic CAPAN-1 cell line overexpressing AP-2*α*. Consequences of overexpression were studied in terms of *in vivo* cell growth, gene expression, migration capacity and chemosensitivity.

**Results:**

*In vivo* tumour growth of CAPAN-1 cells overexpressing AP-2*α* was significantly decreased by comparison to control cells. An altered expression pattern of cell cycle-controlling factors (CDK-4, CDK-6, cyclin-G1, p27^kip1^ and p57^kip2^) was observed in AP-2*α*-overexpressing clones by microarrays and western blot analysis. Promoter activity and ChIP analysis indicated that AP-2*α* induces p27^kip1^ protein levels by direct binding to and transactivation of its promoter. Moreover, AP-2*α* overexpression increased the chemosensitivity of CAPAN-1 cells to low doses of gemcitabine and reduced their *in vitro* migration capacity.

**Conclusion:**

Our data suggested that AP-2*α* overexpression could be exploited to decrease *in vivo* tumour growth of pancreatic cancer cells and to increase their sensitivity to gemcitabine.

Activator protein-2*α* (AP-2*α*) belongs to the AP-2 family of transcription factors that comprises five members encoded by separate genes. All AP-2 proteins share two functional domains, that is, a proline/glutamine-rich transactivation domain and a helix-turn-helix DNA-binding domain. This last one allows AP-2 proteins to bind to GC-rich consensus regions located in the promoters of their target genes (Eckert *et al*, 2005). As recently reviewed (Orso *et al*, 2008), AP-2 proteins have relevant roles in tumorigenesis by regulating some key genes involved in cell proliferation (*ErbB-2*, *c-myc* proto-oncogenes), cell cycle regulation (*CDKN1A*), cell adhesion and invasion (*MMP-2*, *MMP-9*, *PAR-1* and *MUC18*). Moreover, several lines of evidence suggested that AP-2*α* might behave as a tumour-suppressor gene (TSG) in several tissues. For example, loss of AP-2*α* expression was reported in metastatic melanoma cells (Tellez *et al*, 2003), in prostate (Ruiz *et al*, 2001) and colon cancer cells (Schwartz *et al*, 2007). In breast cancer cells, contrasting results were reported in the literature. Earlier studies suggested that AP-2 proteins promote the malignant potential of breast cancer cells by transactivating the promoters of *ErbB-2* and *ErbB-3* (Bosher *et al*, 1996). However, recent data using *sh*RNA to knockdown AP-2*α* expression in breast cancer cell lines showed that AP-2*α* inhibits tumour growth both *in vitro* and *in vivo* (Orso *et al*, 2008). Besides being a potential TSG, AP-2*α* also participates in the control of colon and breast cancer cells sensitivity towards chemotherapeutic drugs (Wajapeyee *et al*, 2005). Despite its potential interest, very few data are available regarding the roles of AP-2*α* in pancreatic cancer (Vernimmen *et al*, 2003). Recently we showed that, in human pancreas (Fauquette *et al*, 2007), AP-2*α* was expressed by almost 66% of non-tumoural ductal cells and endocrine cells by immunohistochemistry, whereas its expression was decreased to only 5.5% of pancreatic ductal adenocarcinoma (PDAC). We also showed that the expression of AP-2*α* was mutually exclusive with that of the human epithelial mucin, MUC4, which is a specific marker of this condition, being expressed in 83% of the PDAC samples. These data suggested that AP-2*α* might also behave as a TSG in human PDAC.

Interestingly, PDAC corresponds to the most frequent histological form of pancreatic cancer, which remains a devastating condition with only 3% of patients alive at 5 years after diagnosis. This is because of the inability to detect cancer at onset, its aggressiveness and the lack of effective therapies (Schneider *et al*, 2005). Indeed, at the time of diagnosis, more than 80% of patients have locally advanced or incurable metastatic disease. Gemcitabine, the only approved drug for the treatment of pancreatic cancer, offers a partial response in <6% of the patients (Chua and Cunningham, 2006). Our interest first focused on the epithelial mucin, MUC4, in this disease because it is not expressed by normal pancreatic tissue but is gradually expressed during pancreatic carcinogenesis from the premalignant lesions, that is, pancreatic intraepithelial neoplasia (PanIN) type 1 (which are MUC4-positive in 17% of the cases) to PDAC (Swartz *et al*, 2002). Moreover, experimental data showed that the knockdown of *MUC4* expression by *sh*RNA in HPAF cancer cell line is accompanied by a significant decrease of cell proliferation *in vitro*, tumour growth and incidence of metastasis *in vivo* (Chaturvedi *et al*, 2007), suggesting that MUC4 could have an important role in pancreatic carcinogenesis. Indeed, MUC4 forms a protein complex with the ErbB-2 receptor in human pancreatic cancer cells and modulates its downstream signalling (Chaturvedi *et al*, 2008).

To decipher the molecular mechanisms that govern *MUC4* expression in pancreatic cancer, we previously characterised its promoter and identified several consensus binding sites for AP-2 transcription factor (Perrais *et al*, 2001). In our recent study, we showed that AP-2*α* is a trans-repressor factor of *MUC4* promoter in two well-differentiated pancreatic cancer cell lines that expressed MUC4. We thus produced several stable clones derived from CAPAN-1 cell line that stably overexpressed AP-2*α*. These clones are characterised by a strong downregulation of *MUC4* along with a strong upregulation of the cdk inhibitor, p27^kip1^ (Fauquette *et al*, 2007). Interestingly, we also showed that these clones exhibited a significant decrease of *in vitro* cell growth and cell invasion. In this study, our aims were to evaluate whether AP-2*α* overexpression could modulate the phenotype of the parental CAPAN-1 cell line in terms of chemosensitivity, migration capacity, pattern of gene expression, topography of epithelial markers expression and *in vivo* cell growth.

## Materials and methods

### Total cellular extract preparation and western blotting

After scraping, the cells were pelleted, washed with 1 × phosphate-buffered saline (PBS) and incubated in lysis buffer (50 mM Tris–HCl pH 7.5, containing 150 mM NaCl, 1% (w/v) NP40, 5 mM sodium fluoride, 5 mM sodium orthovanadate, 0.25% (w/v) sodium deoxycholate and a cocktail of protease inhibitors) for 30 min at 4°C. After 10 min of centrifugation at 13 210 **g** (4°C), the supernatant (total extract) was stored at −80°C. Western blots were carried out as previously described (Fauquette *et al*, 2005). Peroxydase-conjugated secondary antibodies were used and immunoreactive bands were visualised using the West Pico chemoluminescent substrate (Perbio, Brebières, France).

### Confocal microscopy

Cells were fixed with 4% paraformaldehyde (w/v) for 20 min, quenched for 20 min with 50 mM NH_4_Cl in PBS and permeabilised with 0.2%. (w/v) saponin in PBS for 20 min. The saturation step was performed for 20 min in PBS containing 1% bovine serum albumin (BSA) and 0.2% saponin (w/v). Cells were then incubated overnight with different primary antibodies diluted in PBS containing 1% BSA and 0.2% saponin. After PBS washings, cells were incubated for 2 h with secondary fluorescein isothiocyanate or tetra methyl rhodamine isothiocyanate (TRITC)-conjugated antibodies. Laser microscopy analysis was carried out using a Leica instrument (Model TCS-NT) with a 63 × 1.32 Plan-Apochromat oil-immersion objective lens (Leica Microsystems, Cedex, France). Files of microphotographs were processed with Adobe Photoshop 5.0. (San Jose, CA, USA).

### Antibodies

For confocal microscopy analysis, monoclonal antibodies against MUC1 (214D4) and MUC4 (m8G7) were used. These were kind gifts from J Hilkens (The Netherlands Cancer institute, Amsterdam, The Netherlands) and SK Batra (Eppley Cancer Institute, Omaha, NE, USA), respectively. For western blotting, membranes were probed with antibodies against ErbB-2 (NCL CB11 from Novo Castra, dilution 1/250), cyclin-D1 (sc-718, dilution 1/500), cyclin-G1 (sc-7865, dilution 1/500), cdk-4 (sc-601, dilution 1/1000), cdk-6 (sc-177, dilution 1/1000), p57 (sc-1040, dilution 1/2000, all from Santa Cruz Biotechnology Inc., Heidelberg, Germany) or *β*-actin (A5441, dilution 1/5000 from Sigma, St Quentin Fallerier, France).

### Subcutaneous (s.c.) xenografts

CAPAN-1 cells and its deriving clones were injected s.c. (4 × 10^6^ cells in 0.4 ml of RPMI) into athymic female nude mice that were bred and maintained in pathogen-free conditions (six mice per cell type). Tumour development was followed periodically. The tumour volume (mm^3^) was determined by the equation V=W^2^.L/2, in which W corresponds to the width (in mm) and L to the tumour length (in mm). Mice were killed at days 22 or 28 after inoculation. All animal procedures were in accordance with the guidelines of the animal care committee. For each subcutaneous tumour, one tissue fragment was fixed in formalin before inclusion into paraffin.

### Microarrays

To monitor gene expression in response to AP-2*α* overexpression, RNA was prepared from C5, *α*27 and *α*42 clones using Macherey Nagel NucleoSpin RNA II kit (Hoerdt, France). RNA quality was checked using the Agilent 2100 Bioanalyser (IFR 114 facility, Agilent Technologies, Massy, France). The complementary labelled RNA probe was synthesised using the Truelabeling AMP kit (SuperArray Bioscience, SA Biosciences, TebuBio, Le Perray-en-Yvelines, France) and biotin-16-UTP (Roche, Meyba, France), and hydridised with the cell cycle OligoGEArray (SuperArray Bioscience). Signal was generated using the ECF substrate (GE Health care, Orsey, France), and detected using the storm system, and analysed using the GEArray Expression Analysis Suite (SuperArray Bioscience) with normalisation with housekeeping genes.

### Transient transfections

The pancreatic cancer cells, CAPAN-1, were transfected with 0.5 *μ*g of the pGVB2 basic vector carrying promoter elements of p27^kip1^ gene *(CDKN1B)* upstream of the luciferase gene (kind gift from Pr T Sakai, Kyoto Prefectural University of medicine, Kyoto, Japan) as previously described (Fischer *et al*, 2005), and 0.25 *μ*g of an expression vector encoding AP-2*α* (pRSV-AP-2 gift from Dr H Hurst, ICRF Molecular Oncology Unit, London, UK). Results were expressed as fold induction relative to the cotransfection performed in the presence of the corresponding empty expression vector, as previously described (Fauquette *et al*, 2005). Two putative AP-2-binding sites located at −316 and −244 of *CDKN1B* promoter were mutated separately using the QuickChange Site-directed mutagenesis kit (Stratagene, Agilent Technologies, Massy, France) as previously described (Fauquette *et al*, 2005). The −316 site was mutated from 5′-CCGCGGGC-3′ to 5′-TAGCGAAT-3′ and the −244 site from 5′-GCCCTCCCG-3′ to 5′-AATCTCTAA-3′. The mutated plasmid DNA was sequenced on both strands before being used in cell transfection experiments.

### ChIP assay

The Chromatin Immuno Precipitation (ChIP) assay was carried out as previously described (Fauquette *et al*, 2007) using 6 *μ*g of an anti-AP-2*α* antibody (C18 from Santa Cruz Biotechnology) or a normal rabbit IgG (Upstate, Millipore, St Quentin en Yvelines, France). For the PCR, two pairs of primers were designed to selectively amplify a distal (−610/−479) or a proximal region (−426/−20) of the *CDKN1B* promoter. PCR was carried out in a 50 *μ*l volume containing 50 ng of DNA, 5 U of AmpliTaq Gold (Applied Biosystems, Courtaboeuf, France), 0.5 *μ*M of each primer, 2.5 mM MgCl_2_ and 5% dimethylsulphoxide using the following protocol: 3 min at 95°C followed by ((95°C) 15 s, (X_1_1°C per cycle) 15 s, (72°C) 15 s) × 10, ((95°C) 15 s, (X_2_°C) 15 s, (72°C) 15 s) × 32 and 72°C for 5 min, where X_1_ and X_2_ were (58°C, 48°C) for the distal region and (64°C, 55°C) for the proximal region, respectively. PCR products were analysed on a 0.8% agarose gel.

### Effects of gemcitabine *in vitro*

Gemcitabine was a kind gift from Eli Lilly (Indianapolis, IN, USA). Cells were plated in 6-well sterile plates at a 10^5^ cells per well and were allowed to attach overnight. Cells were treated either with 0.001—100 *μ*g ml^–1^ of gemcitabine for 1 h as previously described (Giovannetti *et al*, 2004). After drug treatments were completed, the cytotoxicity was expressed as the percentage of surviving cells relative to untreated cultures. C5 mock cells were used as control cell. Two independent experiments in triplicate were carried out.

### Flow cytometry

Cells were treated with low doses of gemcitabine (0, 0.1 or 1 *μ*g ml^–1^) during 24 h before being harvested by trypsinisation, and then resuspended in PBS. The cells were fixed by addition of 1 ml of 70% ethanol and incubation on ice for 30 min. Cells were then washed with PBS, treated for 5 min by RNAse A (100 *μ*g ml^–1^) and finally stained with propidium iodide (50 *μ*g ml^–1^) for 30 min. Cell analysis was carried out on a Beckman Coulter EPICS XL3-MCL (Villepinte, France) using the Wincycle software (Phoenix Flow Systems, San Diego, CA, USA).

### Migration assay

Cell migration properties of the different clones were assessed using 24-well Boyden chambers (BD Bio Coat Insert 8 pm) with 8.0 *μ*m pores (BD Biosciences, Le Pont de Claix, France) following the manufacturer protocol. Briefly, 15% foetal bovine serum was used as chemoattractant in the lower chamber. A total of 5 × 10^4^ cells were plated in the top chamber and were incubated for 48 h. After staining with Diffquick (Médiane Diagnostics, Plaisir, France), cells on the lower surface were counted on microscopy at × 100 magnification. Eight random vision fields were counted and the experiment was repeated four times.

### Statistical analysis

Data are presented as mean±s.d. Differences in the mean of two samples were analysed using Student's *t*-test with differences <0.05 considered significant.

## Results

### Expression and localisation of MUC1 and MUC4 in CAPAN-1 clones overexpressing AP-2*α*

**Figure 1:**
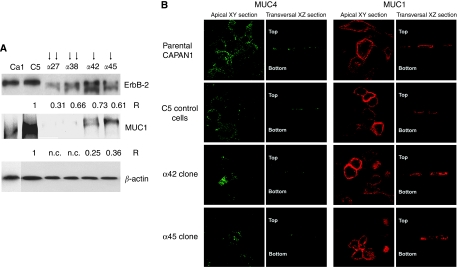
Expression pattern of MUC1 membrane-bound mucin and ErbB-2 receptor in the CAPAN-1 clones stably transfected with an AP-2*α* expression vector (*α*27 to *α*45). (**A**) Western blot analysis of the expression of ErbB-2 and MUC1. One or two upper arrow(s) correspond to a low or high levels of expression of AP-2*α*, respectively. Bands were quantified by densitometry and a ratio (specific protein to *β*-actin) was calculated to evaluate differences between mock cells and the clones. Ratio values are indicated under each panel. Total cellular extracts were used. Ca1, parental cell line; C5, mock cells. (**B**) Topography of MUC1 and MUC4 expression was analysed by confocal microscopy.

We previously produced stable clones deriving from CAPAN-1 cell line that overexpressed moderate (clones *α*42 and *α*45, one arrow on Figure 1A) or high levels (*α*27 and *α*38, two arrows on Figure 1A) of AP-2*α*, and consequently exhibited a partial to complete inhibition of MUC4 expression, respectively (Fauquette *et al*, 2007). To further characterise the pattern of expression of these clones, levels of expression of ErbB-2 (the putative MUC4 receptor) and of the membrane-bound mucin MUC1 were determined by western blotting. As previously shown with MUC4 (Fauquette *et al*, 2007), AP-2*α* overexpression is accompanied by a decrease of the expression levels of total ErbB-2 and MUC1 (Figure 1A). A nearly complete extinction of MUC1 was observed in clones expressing high levels of AP-2*α*. We further examined whether AP-2*α* overexpression could affect the localisation of MUC4 and MUC1 using confocal microscopy analysis. In parental CAPAN-1 cells and in C5 control cells (mock cells), MUC4 was mostly localised at the apical surface, whereas MUC1 showed both apical and basolateral staining (Figure 1B). In *α*42 and *α*45 clones that still expressed membrane-bound mucins, MUC4 apical staining was strongly decreased, whereas MUC1 showed a slight decrease of the apical staining together with an enhancement of the basolateral staining.

### Xenografts of CAPAN-1 cellular clones overexpressing AP-2*α* in nude mice

**Figure 2:**
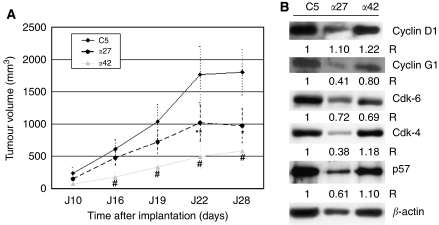
Effect of AP-2α over expression on tumour growth and cell cycle. (**A**) *In vivo* tumour growth of mock CAPAN-1 cells (C5 control clone), and of two clones overexpressing AP-2*α* s.c. xenografted in athymic female nude mice (*n*=6 per group). Tumours were measured every 3–6 days and tumour volume was calculated (see Materials and Methods section for further details). Data are shown as mean (points)±s.d. (error bars). ^*^, *P*<0.05; ^**^, *P*<0.01; #, *P*<0.001. (**B**) Expression levels of regulators of the cell cycle were evaluated by western blotting in control C5, *α*42 and *α*27 clones. Bands were quantified by densitometry and a ratio (specific protein to *β*-actin) was calculated to evaluate differences between mock cells and the clones. Ratio values are indicated under each panel.

Three stable clones derived from CAPAN-1 cell line were s.c. implanted in nude mice (*n*=6 mice per group): *α*27 and *α*42 clones that stably overexpressed high and moderate levels of AP-2*α* transcription factor, respectively, and the control C5 clone. As shown in Figure 2A, the tumour volume was significantly lower in animals xenografted with the *α*27 or *α*42 clones than in those with the C5 clone. At day 22, the mean volume was 1766±433 mm^3^ in controls (*n*=6), 1014±280 mm^3^ in *α*27 (*n*=6; *P*<0.01 *vs* control clone) and 493±189 mm^3^ in *α*42 animals (*n*=6; *P*<0.001 *vs* control clone, *P*<0.01 *vs α*27). On day 28, the difference of growth was significant only between controls (*n*=3) and *α*42 animals (*n*=3; *P*<0.001).

### Analysis of the expression of cell cycle regulators in CAPAN-1-deriving clones

As *α*27 and *α*42 clones differed in terms of *in vivo* cell growth, we decided to compare their transcriptome using oligoGEArrays focused on cell cycle regulators. Results are shown in Table 1. The *α*27 clone exhibited a pattern of expression suggesting an inhibition of the cell cycle progression from the G1 to S phase (decrease of cyclin-D1 and cdk-6 mRNAs) and from the G2 to M phase (decrease of cyclin-G1 cyclin-A1 and cdk-5 protein partner mRNAs). Interestingly, the *α*42 clone exhibited a quite different pattern of expression characterised by an increase of *CDKN1B* (p27^kip1^) and *CDKN1C* (p57^kip2^) mRNAs together with a downregulation of *cyclin*-*D1* mRNA, suggesting a strong cell cycle arrest at the G1/S phase in these cells. We try to validate our microarray data by carrying out western blot analysis for several proteins. We thus confirmed a decrease of CDK-4, CDK-6, cyclin-G1 and p57^kip2^ in *α*27 clone and an increase of p57^kip2^ in *α*42 clone. Previously we showed that p21^cip1^ and p27^kip1^ protein levels were increased in *α*42 clone (Fauquette *et al*, 2007). However, we were unable to confirm a decrease of cyclin-D1 at the protein level in both clones.

### AP-2*α* activates p27^kip1^ promoter

**Figure 3:**
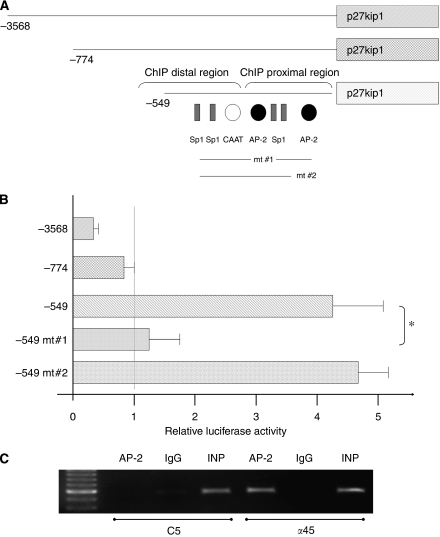
AP-2α activates *CDKN1B* by direct interactions with its promoter. (**A**) Schematic representation of human *CDKN1B* promoter. The main *cis* elements for Sp1, CAAT box-binding factor and AP-2 are shown. The location of the AP-2 *cis* elements that undergo site-directed mutagenesis is also shown. (**B**) The different *CDKN1B* promoter constructs were transiently cotransfected with 0.2 *μ*g of pRSV-AP-2*α* or corresponding empty vector. Results are expressed as fold induction of luciferase activity relative to the empty expression vector (value was set at 1.0). Values are means±s.d. for two independent experiments in which cotransfections were run in triplicate (^*^, *P*<0.05). (**C**) ChIP assay. AP-2 represents the fraction precipitated with the anti-AP-2*α* antibody, IgG those precipitated with a normal rabbit IgG. PCR results obtained on the proximal region are shown.

We previously showed that AP-2*α* overexpression led to an upregulation of p27^kip1^ protein levels in pancreatic cancer cells (Fauquette *et al*, 2007). As p27^kip1^ expression is regulated at transcriptional and post-transcriptional levels, we next wanted to determine whether AP-2*α* could act at the promoter level. To do so, CAPAN-1 cells were transiently cotransfected with a series of three deletion mutants of *CDKN1B* promoter (Figure 3A) along with an AP-2*α* expression vector. As shown in Figure 3B, loss of the −3568 to −774 nucleotides in the *CDKN1B* promoter did not affect AP-2*α* effect that remained weak. Further deletion up to −549 nucleotides allowed AP-2*α* to increase the luciferase activity of the *CDKN1B* promoter activity by 4.3-fold, suggesting the presence of inhibitory *cis* elements in the upstream region. Interestingly the −549/−1 region contains two putative binding sites for AP-2 located at −315 and −243, and we decided to check their implication in AP-2*α* effect by site-directed mutagenesis. Disruption of AP-2 site#1 located at −315 led to a strong reduction of AP-2*α* stimulatory effect (*P*<0.05), whereas disruption of AP-2 site #2 at −243 had no consequence. To decipher what happens *in vivo,* ChIP assay was carried out on two regions of *CDKN1B* promoter, that is, a distal part (−610/−479) that did not contain the two AP-2 *cis* elements mentioned earlier and a proximal part (−426/−20) that did contain these elements. As expected, a strong binding of AP-2*α* to the proximal part of *CDKN1B* promoter was observed in the *α*45 clone that overexpressed the p27 protein (Figure 3C).

### Effect of AP-2*α* overexpression on gemcitabine sensitivity of pancreatic cancer cells

**Figure 4:**
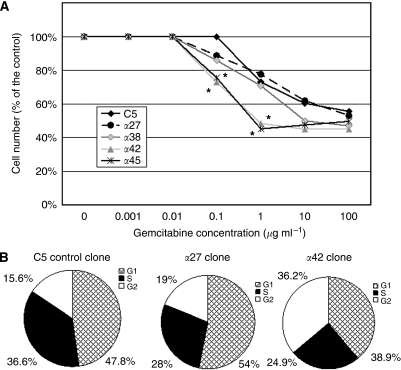
Effect of AP-2α over expression on gemcitabine sensitivity of pancreatic cancer cells. (**A**) *In vitro* growth-inhibitory effect of gemcitabine against the mock CAPAN-1 cells (C5) and the four clones that stably overexpressed AP-2*α* transcription factor. Cell growth was assessed by cell counting of surviving cells. For each cell line, results are expressed as the percentage of surviving cells relative to the untreated cells that were arbitrarily given a 100% value. Results are shown as mean of two independent experiments run in triplicate. ^*^, *P*<0.05. (**B**) Cell cycle distribution profiles of C5 control cells, *α*27 and *α*42 treated with 0.1 *μ*g ml^–1^ of gemcitabine for 24 h. The values are expressed as the mean of three independent experiments.

As the expression level of AP-2*α* was shown to determine the sensitivity of colon or breast cancer cell to chemotherapeutic drugs (Wajapeyee *et al*, 2005), we decided to evaluate whether AP-2*α* overexpression could affect the chemosensitivity of CAPAN-1 cells. A dose-dependent inhibition of cell proliferation was observed with gemcitabine for all the clones tested (Figure 4A). However, only the two clones that overexpressed moderate levels of AP-2*α* (*α*42 and *α*45) exhibited a higher sensitivity to low concentrations of gemcitabine than the C5 control clone (*P*<0.05). In contrast, the *α*27 and *α*38 clones, expressing high levels of AP-2*α* and no MUC4, did not differ from control clone in terms of sensitivity to gemcitabine. Next, the effects of low doses of gemcitabine were further examined using flow cytometry. In absence of gemcitabine, the cell cycle profile of C5 control cells resembled that of *α*27 or *α*42 clones with 41.6±2.3, 48.4±1.4 and 43.4±2.7% in the S phase, respectively (not shown). In contrast, after a 24-h treatment with 0.1 *μ*g ml^–1^ of gemcitabine, a strong cell cycle arrest in the G2 phase was observed in the *α*42 clone by comparison with C5 control clone (*P*<0.05). A trend toward a cell cycle arrest in G1 occurred in *α*27 clone but remained nonsignificant (Figure 4B). Higher doses of gemcitabine (1 *μ*g ml^–1^) led to a G1 phase synchronisation for all clones (not shown).

### Migration assay

**Figure 5:**
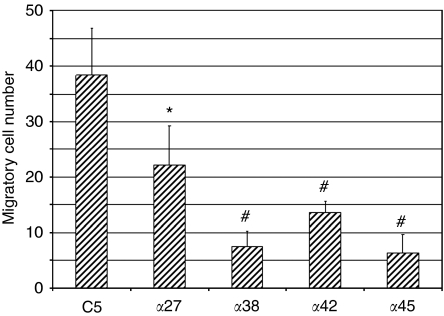
Migration assay. Migration properties of mock CAPAN-1 cells (C5 control clone) and clones overexpressing AP-2*α* (*α*27, *α*38, *α*42 and *α*45) were evaluated using Boyden chambers. Results are expressed as average migratory cell number per vision field (mean±s.e.m., ^*^, *P*<0.05, #, *P*<0.001).

Previous studies conducted in our laboratory showed that stably overexpressed AP-2*α* cells displayed a significant decrease of their invasion capacity *in vitro* (Fauquette *et al*, 2007). In this study, cell migration was evaluated using Boyden chambers. As shown in Figure 5, the number of migrating cells strongly decreases in all overexpressing AP-2*α* cells clones compared with the C5 control clone (*P*<0.05 for *α*27 and *P*<0.001 for *α*38, *α*42 and *α*45 *vs* control C5), suggesting that AP-2*α* has a major role in migration properties of pancreatic cancer cells *in vitro*. Interestingly, *α*42 cells migrated less than *α*27 cells *in vitro* (*P*<0.05) in accordance with their different behaviour *in vivo* (Figure 2A).

## Discussion

In a previous study we showed that AP-2*α* expression concerned only 5.5% of human PDAC tissues, whereas it was expressed by nontumoural pancreatic ductal cells (Fauquette *et al*, 2007). We therefore hypothesised whether such a loss of expression of AP-2*α*, which is considered as a TSG in different tissues (Ruiz *et al*, 2001; Schwartz *et al*, 2007), could contribute to pancreatic carcinogenesis. To address this question, we took advantage of stable clones deriving from CAPAN-1 cells and overexpressing AP-2*α* that we previously generated (Fauquette *et al*, 2007).

In this study, we found that a moderate overexpression of AP-2*α* significantly inhibits pancreatic tumour growth in animal models. Our experimental data, together with recent results obtained on different cell lines (Orso *et al*, 2008), suggested that this anti-tumour activity resulted from a cell cycle arrest as we previously showed that the cyclin-dependent kinase inhibitor p27^kip1^ protein levels in pancreatic cancer cells were strongly increased after an AP-2*α* overexpression (Fauquette *et al*, 2007). Moreover, the growth curves of the tumours deriving from the xenografted clones are inversely correlated to the p27^kip1^ protein levels. We therefore decided to evaluate whether AP-2*α* could regulate p27^kip1^ expression. Here we further showed that AP-2*α* could induce *CDKN1B* promoter activity directly by interacting with two proximal AP-2 *cis* elements located at −316 and −244. Interestingly, the same proximal region of *CDKN1B* promoter was recently shown to contain three *cis* elements for KLF4 transcription factor close to the AP-2 sites and to be induded by KLF4 in pancreatic cancer cell lines (Wei *et al*, 2008). Moreover, KLF4 overexpression led to a suppression of tumour cell growth both *in vitro* and *in vivo*, as shown for AP-2*α*. Whether AP-2*α* and KLF4 could cooperate on *CDKN1B* proximal promoter remains to be defined.

We next try to further document the impact of AP-2*α* on the cell cycle regulators. Microarray analysis and western blotting allowed us to distinguish changes in gene expression linked to AP-2*α* overexpression that were independent (observed in *α*27 clone) or dependent of MUC4 expression (only seen in *α*42 clone). In absence of membrane-bound mucins, AP-2*α* represses the expression of the CDK-4 and CDK-6 that are activated during the G1 phase of the cell cycle (Malumbres and Barbacid, 2009) in presence of their protein partner (cyclin D). These variations are expected to promote a G1 phase arrest in *α*27 clone. Decrease of CDK-4 seems of special interest as its constitutive activation in a mice model results in epithelial tumours affecting the gut, liver and digestive exocrine glands (Sotillo *et al*, 2001). CDK-4 thus has a central role in epithelial proliferation control. In contrast, MUC4 expression reverses the AP-2*α*-induced CDK-4 decrease but, in the same time, is associated with an upregulation of the CDK-I p27^kip1^, p57^kip2^ and p21^cip1^ (Fauquette *et al*, 2007). We therefore speculate that MUC4 could trigger the G1 phase arrest initiated by AP-2*α* overexpression. This result was expected in light of previous data showing that the rat Muc4 interacts with ErbB-2 receptor, that is expressed in *α*42 clone, and induces p27^kip1^ expression (Jepson *et al*, 2002).

We also showed that a moderate overexpression of AP-2*α* in CAPAN-1 cells resulted in an increase of the chemosensitivity to low doses of gemcitabine. This original result, potentially relevant for pancreatic cancer therapy in which gemcitabine is the first-line treatment, was however expected as AP-2*α* was previously shown to increase colon cancer cell sensitivity to chemotherapy. This effect was independent of p53 (Wajapeyee *et al*, 2005) and resulted from a downregulation of Bcl-2 and an induction of apoptosis (Wajapeyee *et al*, 2006). Moreover, the same group also showed that AP-2 was induced by a post-transcriptional mechanism by various chemotherapeutic agents such as adriamycin or cisplatin. However, no data are currently available in the literature to support such an effect for gemcitabine.

Another question to address is whether the decrease of MUC1 and MUC4 expression could contribute to the AP-2*α*-induced sensitisation of pancreatic cancer cells to gemcitabine. Indeed, recent studies showed that MUC1 overexpression in carcinoma cells increases their refractoriness to chemotherapeutic drug-induced apoptosis (Ren *et al*, 2004; Kalra and Campbell, 2007). Accordingly, knockdown of *MUC1* sensitises thyroid cancer cells to doxorubicine through activation of the intrinsic apoptotic pathway (Siragusa *et al*, 2007). Concerning MUC4, its silencing by *sh*RNA in the pancreatic CD18-HPAF cell line led to a significant increase of apoptosis and to an induction of apoptosis mediators, such as caspase-2, caspase-3 and caspase-7, and a repression of the anti-apoptotic protein S100A4 (Chaturverdi *et al*, 2007). However, there is no report in the literature showing that *MUC4* silencing could enhance the cytotoxic action of chemotherapeutic drugs such as gemcitabine. Therefore, we speculate that in our cellular model, a slight decrease of MUC4 expression could potentiate AP-2*α* pro-apoptotic effect. On the opposite, a complete extinction of MUC4 expression seems unfavourable in terms of sensitivity to gemcitabine.

In conclusion, our results suggested that AP-2*α* overexpression could be exploited to decrease the *in vivo* tumour growth of pancreatic cancer cells and probably to increase their sensitivity to conventional chemotherapy.

## Figures and Tables

**Table 1:** Selected genes relative to the cell cycle control whose expression was modified by AP-2*α* overexpression in CAPAN-1 cell line in comparison with the control cells

**Symbol**	**Gene name**	***α*27 clone/C5 clone**	***α*42 clone/C5 clone**
CCNA1	Cyclin-A1	0.31	1.57
CCND1	Cyclin-D1	0.05	0.14
CCNG1	Cyclin-G1	0.39	1.36
CDK4	Cyclin-dependant kinase 4	0.95	1.15
CDK6	Cyclin-dependant kinase 6	0.49	3.70
CDK5R1		0.07	2.11
CDK5RAP3		0.14	3.26
CDKN1A	p21^cip1^	0.32	0.41
CDKN1B	p27^kip1^	0.88	1.67
CDKN1C	p57^kip2^	0.05	2.28
